# Contrasting habitat associations of imperilled endemic stream fishes from a global biodiversity hot spot

**DOI:** 10.1186/1472-6785-12-19

**Published:** 2012-09-26

**Authors:** Albert Chakona, Ernst R Swartz

**Affiliations:** 1 South African Institute for Aquatic Biodiversity, Private Bag 1015, Grahamstown, 6140, South Africa; 2 Department of Ichthyology and Fisheries Science, Rhodes University, P.O Box 94, Grahamstown, 6140, South Africa

**Keywords:** *Sandelia*, *Pseudobarbus*, *Galaxias*, Habitat use, Breede river system, Cape floristic region, Boosted regression trees, Hierarchical partitioning

## Abstract

**Background:**

Knowledge of the factors that drive species distributions provides a fundamental baseline for several areas of research including biogeography, phylogeography and biodiversity conservation. Data from 148 minimally disturbed sites across a large drainage system in the Cape Floristic Region of South Africa were used to test the hypothesis that stream fishes have similar responses to environmental determinants of species distribution. Two complementary statistical approaches, boosted regression trees and hierarchical partitioning, were used to model the responses of four fish species to 11 environmental predictors, and to quantify the independent explanatory power of each predictor.

**Results:**

Elevation, slope, stream size, depth and water temperature were identified by both approaches as the most important causal factors for the spatial distribution of the fishes. However, the species showed marked differences in their responses to these environmental variables. Elevation and slope were of primary importance for the laterally compressed *Sandelia* spp. which had an upstream boundary below 430 m above sea level. The fusiform shaped *Pseudobarbus* ‘Breede’ was strongly influenced by stream width and water temperature. The small anguilliform shaped *Galaxias* ‘nebula’ was more sensitive to stream size and depth, and also penetrated into reaches at higher elevation than *Sandelia* spp. and *Pseudobarbus* ‘Breede’.

**Conclusions:**

The hypothesis that stream fishes have a common response to environmental descriptors is rejected. The contrasting habitat associations of stream fishes considered in this study could be a reflection of their morphological divergence which may allow them to exploit specific habitats that differ in their environmental stressors. Findings of this study encourage wider application of complementary methods in ecological studies, as they provide more confidence and deeper insights into the variables that should be managed to achieve desired conservation outcomes.

## Background

Knowledge of species specific ecological requirements is a prerequisite for successful conservation [1,2], and for understanding biogeographic and phylogeographic patterns of extant taxa [3,4]. Information on determinants of biodiversity patterns in ecological studies has often been derived from traditional regression methods [5]. Generally, most of these methods focus on identifying the single best model, not on quantifying the independent explanatory power of the predictor variables, yet the latter is likely to provide important insights into the variables that should be managed to achieve desired conservation outcomes. Further, the performance of traditional regression methods is influenced by multicollinearity of explanatory variables as well as by outliers and missing data. These problems may result in the exclusion of ecologically more causal variables from the models [6], thus potentially biasing the actual relationships between species distributions and the environment.

There are a number of alternative statistical approaches that have been developed to improve predictive performance and provide reliable identification of explanatory variables that have the strongest influence on species distribution patterns. These techniques include hierarchical partitioning [6-8], variance partitioning [9] and boosted regression trees (BRT) [10]. The ability of partitioning methods to address the problem of multicollinearity makes them more desirable approaches for ecological studies, because explanatory variables are often only nominally independent. BRT is a relatively new approach for modelling species-environment relationships [10]. Advantages of BRT models include superior predictive performance compared to most traditional modelling methods, ability to handle different types of explanatory variables (data can be categorical, numeric or binary), ability to accommodate missing data, and they do not require elimination of outliers or prior data transformation [10]. BRT models are insensitive to differing scales of measurement, and they can fit complex nonlinear relationships and interactions between predictors [10].

Despite the additional insights that may be gained from partitioning methods and boosted regression trees, these approaches have rarely been applied to the analysis of ecological data [11-15]. The present study applied BRT and hierarchical partitioning to provide insights into the important variables that influence the distribution of stream fishes from the Cape Floristic Region (CFR) of South Africa. The CFR is a hotspot for endemic freshwater biota [16-18]. This region’s high degree of endemism is thought to have resulted from its long period of isolation and complex evolutionary history, which promoted *in situ* diversification [18]. However, the majority of the native stream fishes of the CFR rank amongst the most imperilled freshwater taxa in southern Africa [19]. Nearly all native freshwater fishes of the CFR are already listed in threatened categories of the IUCN, because their historical distributions have declined as a result of multiple anthropogenic impacts, mainly hydrological modifications, degradation of habitats and widespread invasion of the rivers by at least 15 alien fish species [19-22]. These impacts have collectively resulted in several local extinctions in a number of mountain tributaries and extirpation of almost all main-stem populations of native freshwater fishes [20]. The remaining native fish populations persist only in undisturbed headwater tributaries, often above in-stream physical barriers that prevent upstream migration of alien invasive fishes.

Detailed understanding of natural variation of species is essential for predicting past distribution patterns [23], assessing conservation status [24], projecting potential impacts of environmental changes [25], designing and prioritizing conservation areas and formulating recovery programs for threatened species [26]. Such information should best be generated from undisturbed or minimally disturbed systems [27]. The near-natural condition of upland tributaries of the Breede River system in the south-western CFR offered a unique opportunity to study the factors that influence the distribution of stream fishes in the absence of major confounding impacts such as pollution, sedimentation and alien fishes. The Breede River system was previously thought to contain only four indigenous primary freshwater fishes, currently *Galaxias zebratus*, *Pseudobarbus burchelli*, *Sandelia capensis* and *Barbus andrewi*[28,29]. Molecular studies have, however, discovered four deeply divergent genetic lineages within *G. zebratus*, three historically isolated lineages within *P. burchelli* and three lineages of *S. capensis* in the Breede River system [30-32], Chakona *et al*., in preparation. Taxonomic revision of these groups is underway and some of the lineages will be described as distinct species. This study assessed one lineage of *Galaxias zebratus*, one of *Pseudobarbus burchelli* and two lineages of *Sandelia capensis* that co-occur in a number of undisturbed or near-natural mountain tributaries of the Breede River system. *Galaxias* ‘nebula’ (~ 75 mm total length (TL)) has a slender body form and *Pseudobarbus* ‘Breede’ (~ 135 mm TL) is fusiform with forked caudal fins. The two *Sandelia* spp. lineages (~ 200 mm TL) are genetically closely related and have laterally compressed body form. They were therefore combined in all analyses and comparisons. *Barbus andrewi* was not included in the present study because it was only found at two riverine localities. This species now persists in two man made dams in the Breede River catchment.

Specifically, the study addressed three questions: (i) what are the main environmental determinants of the spatial distributions of stream fishes in the CFR? (ii) are there differences in the main determinants of distribution among species? (iii) are the results of BRT and hierarchical partitioning in concordance?

One potential source of differences in the distribution patterns and environmental relationships between stream fishes is differing body morphologies. Freshwater fishes exhibit high morphological divergence, suggesting that they evolved to exploit specific habitats that differ in their environmental stressors [33,34]. The fishes considered in this study have distinct body forms [Additional file 1. It was hypothesised that *Sandelia* spp. would be mainly associated with lower river reaches because fishes with laterally compressed bodies are generally adapted to life in slow flowing waters [35]. *Pseudobarbus* ‘Breede’ were predicted to be capable of exploiting stream reaches with faster flowing waters because fishes with forked tails are generally considered to have improved swimming performance [36,37]. *Galaxias* ‘nebula’ were hypothesised to be capable of exploiting reaches at higher elevation because anguilliform and slender bodied fishes are expected to have reduced energetic expenditure necessary to maintain position in faster flowing water [34,36].

## Results

*Galaxias* was the most widespread lineage, occurring in 61% of the sampled sites and 73% of the streams. *Pseudobarbus* was common and present in 57% of the sampled sites and 62% of the streams. *Sandelia* was uncommon and was present at only 28% of the sampled sites and in 43% of the streams.

### Species distribution and environmental relationships

#### Boosted regression trees

**Figure 1:**
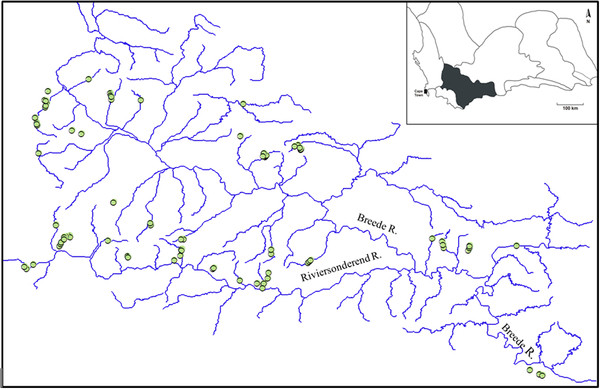
**Species response curves.** Functions fitted for the most important predictors by a boosted regression trees (BRT) model relating the probability of occurrence of *Sandelia* spp., *Pseudobarbus* ‘Breede’ and *Galaxias* ‘nebula’ to environment.

**Figure 2:**
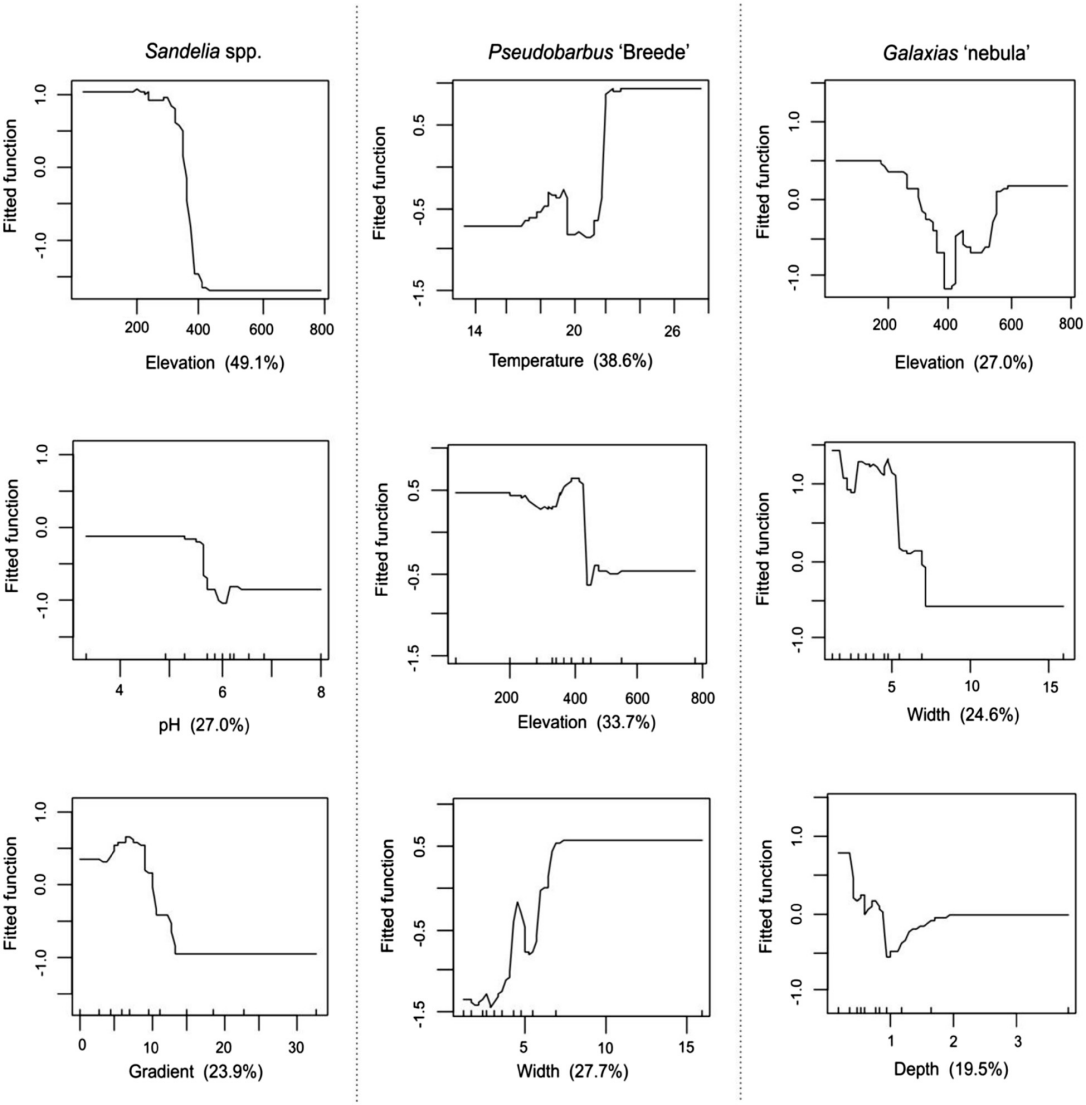
**Probability plot of *Sandelia *spp. occurrence.** Plot of the interaction between slope and elevation showing the predicted probability of occurrence of *Sandelia* spp.

The simplification procedure indicated that elevation, pH and slope were the strongest correlates of *Sandelia* spp. distribution. Elevation contributed almost half of the variation (49.1%), while the relative contributions of pH and slope were well balanced and equal to 27.0% and 23.9%, respectively (Table 1). Model evaluation using 10-fold cross validation suggested very good predictive performance (AUC = 0.88), with a predictive deviance of 33% (Table 2). Fitted functions from the BRT models indicated that reaches located below 400 m and gentle gradients (< 15 m/km) were the most suitable for *Sandelia* spp. (Figure 1). *Sandelia* were frequently caught in reaches with low pH (< 6). Strong interactions were found between elevation and gradient (50.9), as well as between elevation and pH (32.9). There was a pronounced peak of *Sandelia* spp. occurrence in reaches that combined both low elevation and gentle gradient (Figure 2).

**Table 1:** Independent explanatory power of predictors

**Predictor**	***Sandelia*spp.**	***Pseudobarbus*‘Breede’**	***Galaxias*‘nebula’**
Elevation	49.1	33.7	27.0
Width		27.7	24.6
Slope	23.9		14.4
Temperature		38.6	
pH	27.0		
Depth			19.5
Conductivity			14.4

Relative contributions (%) of the predictors for boosted regression trees models relating the distributions of *Sandelia* spp., *Pseudobarbus* ‘Breede’ and *Galaxias* ‘nebula’ to the environment

**Table 2:** Boosted regression trees model performance

**Species**	**No. of trees**	**Deviance**				**Correlation**		**AUC**	
		**Null**	**Residual**	**se**	**percent**	**Cor**	**se**	**auc**	**se**
*Sandelia*	2450	1.193	0.804	0.101	32.6%	0.620	0.081	0.883	0.036
*Pseudobarbus*	3100	1.368	0.971	0.064	29.0%	0.620	0.044	0.848	0.025
*Galaxias*	3200	1.333	1.203	0.060	9.8%	0.380	0.065	0.700	0.040

Predictive performance of models for *Sandelia* spp., *Pseudobarbus* ‘Breede’ and *Galaxias* ‘nebula’. The final number of trees fitted, the null deviance, and cross-validated estimates and standard errors (se) of the predictive deviance, correlation, and area under the receiver operator characteristic curve (AUC) are given for the taxa

**Figure 3:**
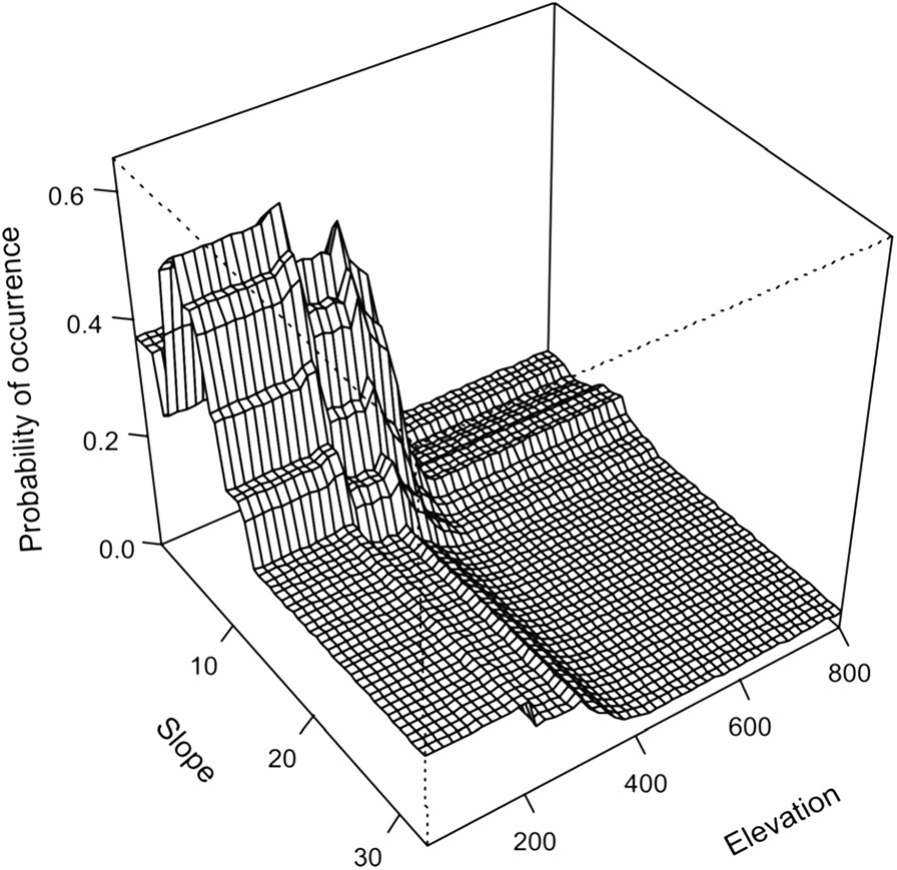
**Probability plot of *Pseudobarbus *‘Breede’ occurrence.** Plot of the interaction between width and temperature showing the predicted probability of occurrence of *Pseudobarbus* ‘Breede’.

For *Pseudobarbus* ‘Breede’, the simplification procedure retained temperature, elevation and width as the most influential predictors, and model evaluation suggested very good predictive performance to independent data (AUC = 0.85) (Table 2). The relative contributions of these variables were 38.6%, 33.7% and 27.7%, respectively (Table 1). Fitted functions from the BRT model indicate that *Pseudobarbus* ‘Breede’ occurred most frequently in wider stream reaches at elevations below 500 m, and temperatures above 20°C (Figure 1). The strongest interaction was found between width and temperature (183.2). Interactions fitted for *Pseudobarbus* ‘Breede’ indicate that this species occurs most frequently in wider stream reaches, but this response is strongly affected by temperature, with higher probabilities of detection in reaches with warmer temperatures (Figure 3).

**Figure 4:**
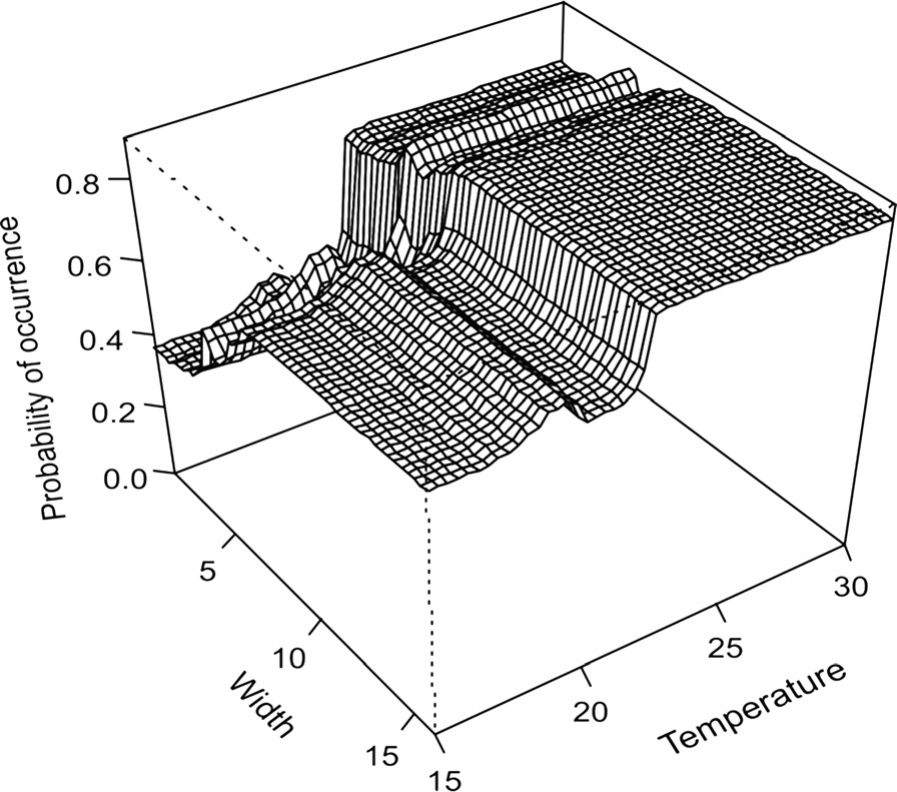
**Probability plot of *Galaxias *‘nebula’ occurrence.** Plot of the interaction between depth and width showing the predicted probability of occurrence of *Galaxias* ‘nebula’.

The simplification procedure indicated that *Galaxias* ‘nebula’ distribution was most strongly influenced by elevation (27%), width (24.6%), depth (19.5%), with slope and conductivity contributing 14.4% each (Table 1). The final model had a predictive deviance of 10% and an AUC of 0.70 (Table 2), indicating fair or useful predictive performance to independent data. Similar to both *Sandelia* and *Pseudobarbus*, fitted functions from the BRT models were non-linear and complex for *Galaxias*. These functions indicate that *Galaxias* ‘nebula’ occurred in a wide range of elevation, but rarely occurred in elevations between 400 and 500 m above sea level. *Galaxias* ‘nebula’ demonstrated a distinct preference for streams up to 6 m wide, and occurred more frequently in reaches with shallow water (< 1 m) (Figure 1). The strongest interactions were found between width and depth (17.0) as well as width and conductivity (16.9). There is a pronounced peak of occurrence of this species in stream sections that combine narrow widths and shallow depths (Figure 4).

#### Hierarchical partitioning

The independent effects of six of the seven variables included in hierarchical partitioning analyses were statistically significant for at least one of the species (Table 3). The total independent contributions (*I*) for all three species were substantially larger than their joint contributions (J). The |*I*/*J*| ratios were 13.5 for *Sandelia* spp., 5.5 for *Pseudobarbus* ‘Breede’ and 5.6 for *Galaxias* ‘nebula’ (Table 4). Similar to the BRT results, hierarchical partitioning also indicated that elevation (27.1%), pH (19.6%) and slope (14.8%) were the most important explanatory variables for *Sandelia* spp. Hierarchical partitioning, however, also revealed that depth (17.7%) had an important independent effect of the distribution of *Sandelia* spp. Width (30.1%) and temperature (29.0%) were the most important explanatory variables for the distribution of *Pseudobarbus* ‘Breede’. Width (42.7%) and depth were the most important explanatory variables for *Galaxias* ‘nebula’. Similar to BRT results, conductivity did not appear to have a very important independent effect on the distribution of the species considered in this study (Table 4).

**Table 3:** Randomisation tests for predictor variables

	***Sandelia* spp.**	***Pseudobarbus* ‘Breede’**	***Galaxias* ‘nebula’**
pH	3.37*	−0.17	−0.63
Temperature	1.11	4.27*	0.30
Conductivity	0.84	1.35	0.93
Depth	2.40*	1.06	0.95
Width	0.09	5.26*	3.83*
Gradient	4.23*	0.33	−0.06
Elevation	5.83*	2.72*	−0.70

Results of the randomization tests for the independent contributions of separate predictor variables in hierarchical partitioning in explaining variation in the distribution of *Sandelia* spp., *Pseudobarbus* ‘Breede’ and *Galaxias* ‘nebula’. (Results are expressed as *Z*-scores. Significant variables (p < 0.05) are indicated with an asterisk)

**Table 4:** Joint and independent effects of predictors

	**Total partition**				***%I***_***i***_						
**Species**	***R***	***I***	***J***	**|*I/J*|**	**WID**	**TEMP**	**DEP**	**ELE**	**SLO**	**CON**	**pH**
*Sandelia*	−91.1	−84.8	−6.3	13.5	3.8	9.4	17.7	27.1	14.8	7.6	19.6
*Pseudobarbus*	−66.9	−81.6	14.7	5.5	30.8	29.0	8.2	14.1	5.3	10.2	2.4
*Galaxias*	−27.4	−33.4	6.0	5.6	42.7	13.1	19.2	0.9	10.7	11.1	2.3
Mean effect					25.8	17.2	15.0	14.0	9.9	9.6	8.4

Decomposition of the total reduction in deviance (*R*) associated with the seven environmental variables into independent (*I*) and dependent (*J*) components of *R* using the hierarchical partitioning method. The independent contribution of variable *I* (%*I*_*i*_) is given for each taxon

## Discussion

### Determinants of species distributions

Elevation, slope, width, depth and temperature were identified as having the most important contribution to the distribution of the studied stream fishes. These results are consistent with studies from other regions showing that ecological boundaries of stream fishes are strongly influenced by elevation, slope and stream size [38,39]. Winemiller et al. [34] documented changes in fish diversity and distributions associated with the altitudinal gradient of streams. A similar effect of altitude has also been reported for the spatial variation in Andean stream fish assemblages [40]. Buisson et al. [13] indicated that elevation and temperature had a strong effect on the spatial distribution of fishes in south-western France, while Amadio et al. [41] have documented the central role of water temperature in determining the upstream boundaries of saugers (*Sander canadensis*) in North America. Temperature was also found to be an important explanatory variable related to the distribution of fish communities inhabiting mountain tributaries of the central Andes in Colombia [40].

The effect of riparian and aquatic vegetation, stream physical structure and bottom cover in influencing the distribution of stream fishes is also important [42]. Riparian vegetation provides shade and allochthonous organic debris, which is an important source of carbon and energy [43], while aquatic vegetation provides in-stream cover and increases habitat diversity. Results from boosted regression trees in the present study, however, indicated that riparian and aquatic vegetation, substrate type and bottom cover were not relevant in explaining the distribution of the fishes considered in the present study. These variables were dropped from all species models by the recursive feature elimination procedure, which excludes non-informative predictors [10]. A possible reason why riparian vegetation may not be important in the mountain tributaries considered in the present study could be related to the low retention time of allochthonous organic debris due to the swift flowing nature of the streams as well as the occurrence of spates during the rainy season. None of the streams were found to have accumulated organic debris. Aquatic vegetation is also largely lacking in most of the undisturbed streams. The relatively low variation in stream physical structure (predominantly cobbles and boulders) could be the reason why substrate type was non-informative in this study. Thus, while some ecological patterns of stream fishes may be common among different geographic regions, some patterns may also be specific to particular regions. This makes it difficult to make broad ecological generalisations.

### Comparative species responses

The distributions of *Sandelia* spp., *Pseudobarbus* ‘Breede’ and *Galaxias* ‘nebula’ were not determined by the same environmental factors. The upstream boundaries of *Sandelia* spp. in mountain tributaries of the Breede River system were strongly affected by elevation and slope. *Sandelia* were not found in reaches that had channel slopes greater than 15 m/km and elevation higher than 425 m, and were primarily associated with pools. Slope and topography affect the distribution of stream animals through their influence on the geomorphology and flow dynamics [44]. High elevation streams and steep gradients are characterised by strong currents and turbulent flow, and this selects for species that have adaptations for maintaining position in fast flowing waters. The observed habitat selection for *Sandelia* may be related to morphological specialisation. *Sandelia* have laterally compressed bodies, large pectoral fins and lower caudal fin aspect ratios (more square shaped caudal fins) [Additional file 1. Studies have shown that fishes with these morphological characteristics have poor swimming performance due to high drag penalties [32]. It is therefore likely that *Sandelia* may not be capable of maintaining position under greater turbulence, due to increased energetic demands. This may explain why *Sandelia* was absent from reaches at higher elevations and steeper gradients. Similar patterns of habitat segregation associated with body morphology have been reported for tropical and neotropical stream fishes [34,40].

Water temperature and mean width (used here as a proxy for stream size) were identified as the primary determinants of *Pseudobarbus* ‘Breede’ distribution. Temperature is considered to be a major ecological factor that directly affects behaviour, metabolism, reproduction, development and growth of freshwater fishes [45-48]. The interaction between stream size and water temperature indicated that the probability of occurrence of *Pseudobarbus* ‘Breede’ was highest in wider reaches and higher temperatures (> 25°C). This pattern may be related to the dietary requirements of this species. The sub-terminal mouth in this species is suited for scraping periphyton or picking small animals from rock surfaces, a behaviour that has been commonly observed during field surveys. Thus, the strong relationship between occurrence of *Pseudobarbus* ‘Breede’ with wider streams and higher temperatures could indicate that this species selects habitats in which environmental conditions promote increased primary and secondary productivity. This pattern is concordant with that reported by Angermeier & Karr [49] who found strong relationship between stream fish biomass and habitat features (e.g. stream size) that maximise the availability of preferred dietary items (reviewed by Winemiller et al. [34]). Although elevation was found to be less influential on the distribution of *Pseudobarbus* ‘Breede’, it is important to note that this species was found to be capable of utilising habitats at higher elevation and steeper gradients (and hence faster current velocities) compared to *Sandelia*. *Pseudobarbus* has a fusiform body shape and higher caudal fin aspect ratio (forked tails) [Additional file 1, two traits that are known to reduce drag and increase swimming ability (thrust) in faster-flowing water [36,37].

Stream size and depth were selected by both boosted regression trees and hierarchical partitioning as important determinants of *Galaxias* ‘nebula’ distribution. The interaction between these two variables indicated that the probability of occurrence of this species was highest in smaller streams and shallow habitats. This agrees with findings from tropical systems where many small stream fishes are associated with smaller streams and shallow habitats that provide refugia from piscivores [34]. *Galaxias* ‘nebula’ occurred at diverse elevations and a wider variety of slopes, and penetrates into higher elevations compared to *Pseudobarbus* ‘Breede’ and *Sandelia* spp. *Galaxias* ‘nebula’ is ecologically adapted to utilise regions of high flow velocity and turbulence. This species has a more slender, cylindrically shaped body and smaller pectoral fins, features that reduce hydrodynamic drag and hence reduces the energetic demands of maintaining position in flowing water [34,36,37]. Species with these traits usually have better swimming performance and are capable of exploiting river reaches with faster current velocities [34]. The regression tree model indicated that *Galaxias* 'nebula' had reduced frequency of occurrence at elevations between 300 and 500 m above sea level. This pattern is not readily explainable, but it may possibly reflect the role of other factors (including biotic interactions) that have not been considered in this study [34].

### Methodological aspects

Minor differences were found between results from boosted regression trees and hierarchical partitioning for some habitat variables. For example, the relative contributions of elevation, slope and pH in explaining the distribution of *Sandelia* spp. were higher in the boosted regression trees than the hierarchical partitioning results. This difference could be related to that fact that data for some of the variables were transformed prior to hierarchical partitioning analyses, while boosted regression trees analyses do not require data transformation prior to analysis. Nevertheless, these two approaches were complementary. Hierarchical partitioning addresses the problem of multicollinearity among predictor variables, but a major weakness of this approach is the inability to account for non-monotonous functions, yet nonlinear responses are quite common in species-environment relationships [38]. Hierarchical partitioning also does not provide information about the type of responses, because its purpose is not to generate a predictive model [50]. The boosted regression trees method was an appropriate alternative to addressing these shortcomings, because it has the ability to fit nonlinear responses between species and environmental predictors [10]. Additional advantages of this approach include the capacity to determine the strengths of interactions between predictors, and fitting the interaction effects to identify optimal habitats for the species [10]. Thus, the simultaneous application of boosted regression trees and hierarchical partitioning in this study helped to identify the predictors that were selected by both methods as the most likely causal variables as well as fitting species responses to them. This provides confidence and deeper insights into the variables that need to be targeted and managed to achieve desired conservation outcomes.

Evaluation of model performance using AUC revealed some differences among the species. Both *Sandelia* and *Pseudobarbus* had substantially high AUC scores than *Galaxias*. *Sandelia* and *Pseudobarbus* also had the highest explained deviance compared to *Galaxias*. A possible explanation why *Galaxias* ‘nebula’s model obtained poor explanatory performance measures could be related to its occurrence in diverse habitats compared to the other species. Alternatively, this may indicate that other factors that were not considered in this study (for example biotic interactions) could be influential in the distribution of *Galaxias* ‘nebula’.

### Conservation implications

The species-specific spatial patterns and environmental relationships found in this study, and also reported from other studies [13,34,38,40], suggest that stream fishes may respond differently to specific impacts, with some species being potentially more vulnerable than others. The invasion of river landscapes in the CFR by alien species and habitat degradation are considered to be the greatest threats to the freshwater biodiversity of this region [19-22]. The restriction of the remnant populations of *Sandelia* spp. to lower sections of mountain streams exposes these lineages to multiple impacts, which include increased susceptibility to invasion by alien predators from the main-stems, hydrological alteration and habitat loss due to building of water abstraction structures in upper reaches, sedimentation and increased water turbidity, pollution and pesticides from intensive agricultural activities. The two *Sandelia* lineages are therefore arguably the most threatened of the fishes considered in the present study. The inclusion of these lineages into one widespread species that was considered to be capable of exploiting diverse habitats [29] clearly masked the real threats to these taxa. Conservation strategies in many data deficient regions has had to rely almost exclusively on expert knowledge, but apart from the implications of cryptic diversity, lack of detailed knowledge of species ecology may misdirect conservation prioritisation, and can potentially lead to loss of biodiversity. For example, the building of weirs to prevent upstream migration of alien species has been considered to be one of the best conservation strategies to secure the remaining populations of threatened fish species [51,52]. Given the species-specific habitat associations of stream fishes, it is clear that careful selection of the location of such barriers is required so that the protected river sections will encompass optimal habitats for all the target species.

Field surveys indicate that *Sandelia* and *Pseudobarbus* have been extirpated from tributaries where weirs have been built at higher altitude. In some instances, the remaining populations only occur in a very short stretch of river above the weirs. This indicates that these weirs have been built just below the fishes’ upper limits. Long-term persistence of these populations is uncertain, because the remaining habitat may not be optimal, and loss of genetic diversity may occur since migration from elsewhere could be blocked by the man-made structures. In most cases, the reaches below water take-off points are completely dry during the summer period, or if water is present, the habitats have been invaded by alien fishes. Given the socio-economic importance of farming and irrigation in the region, complete exclusion of water abstraction from the streams is not feasible. Conservation authorities should therefore seek support from local landowners whose properties have streams that still hold viable populations of native fishes, to ensure that (1) water take-off points and weirs are placed as low as possible in tributary streams, but above alien fish distributions, (2) in-stream habitats are rehabilitated and protected and (3) ecological flows are restored in stream sections that benefit indigenous fishes.

Translocation to undisturbed habitats has been suggested as a useful strategy in the recovery of threatened species [53]. However, in many regions (including the CFR), the remaining undisturbed streams are confined to high altitude mountain catchments where human development is still minimal. Given the species-specific ecological boundaries presented in this study, and also documented for stream fishes from other regions [13,34,38,40], translocation into high altitude streams may not help certain species, while at the same time potentially impacting on other aquatic biota. For example, moving species with laterally compressed bodies such as *Sandelia* into reaches above their natural upstream boundaries may not be a viable long-term conservation measure, since such species are associated with lower river reaches with gentle gradient and an abundance of pools with slow flow. Findings from this study suggest that *Sandelia* could be used as umbrella species [54], because successful protection and restoration of their optimal habitat will indirectly protect other broadly co-distributed freshwater taxa.

## Conclusions

The contrasting habitat relationships of *Sandelia* spp., *Pseudobarbus* ‘Breede’ and *Galaxias* ‘nebula’ support findings from earlier studies that also reported species specific responses of stream fishes to environmental descriptors [13,38]. The species-specific modeling approach used in the present study provides deeper insights into species-environment relationships compared to the use of synthetic descriptors, such as guilds or species richness [55,56]. The use of boosted regression trees and hierarchical partitioning allowed accurate identification of the most influential environmental predictors and the responses of the species to them. Results from the present study are consistent with previous research on stream fishes that suggest strong relationships between fish morphology and ecology [34-37]. These species-specific responses should be considered in conservation planning and management.

## Methods

### Study area

The geology of the CFR is dominated by the Cape Supergroup which consists of extensively folded Table Mountain, Witteberg and Bokkeveld Groups [57,58]. The Bokkeveld Formations are marine deposits and the rivers draining these rock types have high conductivity and high salt content. Erosion of the more resistant Table Mountain Formations produce highly leached quartzite sandstones and the rivers draining these formations have low conductivity and oligotrophic waters. The climate is Mediterranean with dry summers and wet winters resulting from orographic rainfall. Undisturbed rivers in this region have perennial flow.

### Fish distribution data

**Figure 5:**
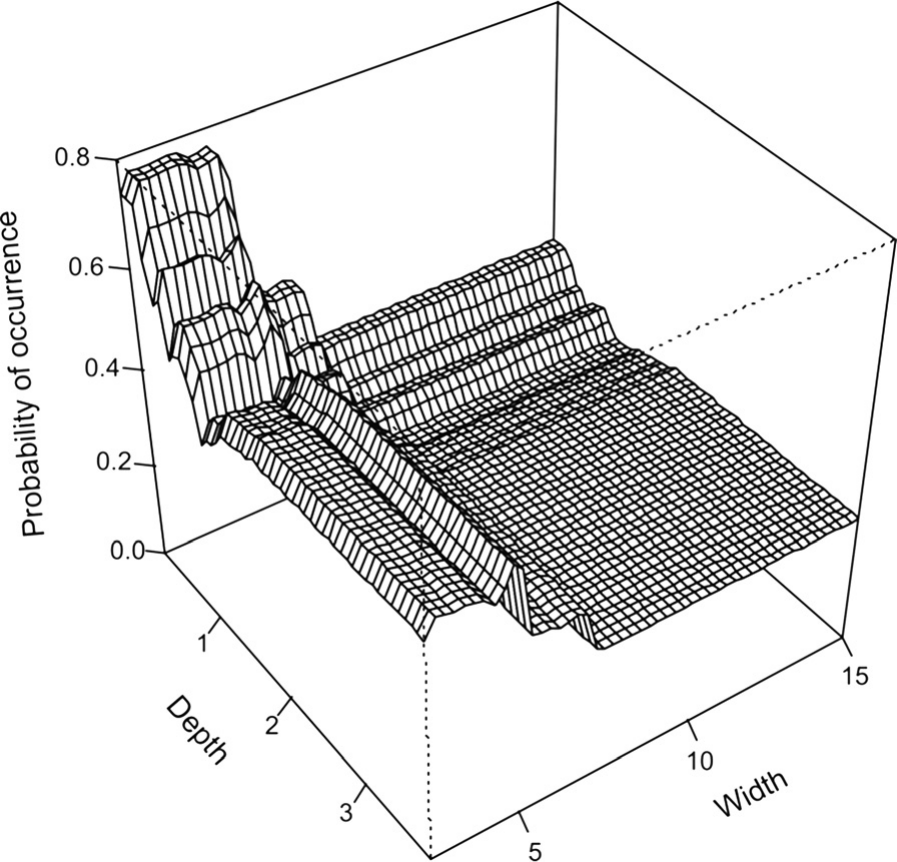
**Map of study area.** Location of sampling sites across the Breede River system. Insert shows location of the Breede River system in the Cape Floristic Region at the southern tip of Africa.

The research was conducted under permit from CapeNature (permit number: AAA-004-000205-0035) issued only after the approval of methods by a review panel. Intensive sampling was conducted during low-flow conditions between November 2008 and December 2009. Data from 148 sites from 44 undisturbed mountain tributaries of the Breede River system (Figure 5) were used in this study. Sites were classified as undisturbed and included in the present study if they were: (i) located upstream of weirs and water diversion structures, (ii) located upstream of agricultural or residential areas, (iii) not invaded by alien fishes and (iv) not isolated by apparent fish barriers (e.g. waterfalls). This was done to ensure that the distribution of the fishes was based on intrinsic habitat preference and not influenced by anthropogenic disturbance, alien fish impacts (predation or competition) or exclusion by natural or artificial barriers to dispersal.

At each locality riffles and pools in a stream section of about 30–50 m were sampled. Due to the relatively small size of the streams, this length usually included more than 3 pool-riffle sequences, which is considered to be adequate for getting a representative sample of fish communities within a reach [59]. Sampling techniques varied depending on the size of the stream, depth and water clarity. Electrofishing (SAMUS-725MP) was used for sampling in shallow riffle stream sections with cobble-boulder substratum, while the occurrence of fish in pools with clear water was determined by snorkelling. Deep tannin-stained pools were sampled with a seine net (3 m length, 3 mm mesh size). While there may be advantages in considering fish densities at each station rather than their presence or absence alone, the present study encompassed a wide geographic region. Time and resource constraints precluded assessment of fish densities at the localities sampled. Therefore the presence-absence approach was used for the present study. Fish at each site were either observed or captured using the methods described above. Captured fish were identified and quickly returned to the water alive, but at some of the localities some fish (up to 10 individuals per species per tributary) were retained for tissue samples for genetic analysis (Chakona et al., unpublished). The location of each sampling site was recorded with a hand held Global Positioning System (GPS) unit with accuracy within 10 m.

### Environmental predictors

Studies from disparate regions have indicated that the distribution of stream fishes is influenced by a number of environmental factors, such as stream size, water depth, flow velocity, substrate types, water temperature and chemistry, riparian and aquatic vegetation, elevation and channel slope [27,38-41]. At each sampling locality, habitat was characterised by quantitative and qualitative measurements of 11 environmental variables. Portable electronic meters were used to measure temperature and conductivity (Hanna EC/TDS/Temperature Tester, HI98311 (DiST 5)) and pH (Hanna pH/Temperature tester HI98128). Local habitat features were characterised by measuring channel width, depth, assessing bottom substratum and aquatic vegetation. Within each reach, 4 to 8 transects were measured for physical habitat variables. Depth was measured with a graduated pole at three equally spaced intervals for each transect. Maximum depth was the greatest water depth measured among transects. Transect widths were used to calculate mean width (used here as proxy for stream size) for each sampling locality. Dominant substratum was visually estimated and characterised as silt-sand (< 2 mm), gravel (10 – 64 mm), cobble (64–256 mm), boulders (> 256 mm) and bed rock (solid rock surfaces) [60,61]. Bottom cover, presence of aquatic and terrestrial riparian vegetation were visually assessed and characterised as none (0), scarce (< 30%), moderate (30 – 60%) and abundant (> 60%). Elevation and channel slope for each site were calculated from GPS coordinates using GIS Spatial Analyst.

### Statistical modelling

#### Boosted regression trees

Boosted regression trees (BRT) was used to determine the relationship between fish occurrence and the 11 environmental variables. A detailed overview of boosted regression trees and guidelines for using this approach are given by Elith et al. [10]. BRT analyses were carried out in the R statistical package version 2.15.1 [62] using the ‘dismo’ library following Elith & Leathwick [63]. Because of the binary nature of the response variable (presence/absence), the binomial error distribution and a logistic link function were used. Tree complexity (*tc*) and learning rate (*lr*) were altered to determine optimal settings for the base model containing all the descriptors. Ten-fold cross validation was used for each optimisation trial, with a random subset of 50% of the data being used to fit each new tree. This was followed by the recursive feature elimination procedure which was used to simplify the base model by dropping non-informative predictors [10]. Predictive performance of the final model was then evaluated using the cross-validation process internal to the model building procedure. This evaluation was based on predictions to sites that were withheld from model fitting. Two performance metrics were determined for each model. Predictive deviance (expressed as a percentage of the total deviance) provides a measure of the goodness-of-fit between predicted and raw values. The second metric is the area under the receiver operator characteristic curve (AUC) which estimates the degree to which fitted values discriminate between observed presences and absences. Values of AUC range from 0.5 to 1.0. Values of AUC > 0.90 indicate excellent distinction between presences and absences, 0.80 - 0.90 is considered very good, 0.70 – 0.80 indicates fair performance, values > 0.60 are considered useful and values < 0.60 indicate poor performance [64-66]. The relative contribution (%) of the individual predictors was evaluated, and environmental optima for each species were determined by plotting the distribution of fitted values in relation to each of the predictors following Elith & Leathwick [63]. The effect of interactions between predictors was also evaluated.

#### Hierarchical partitioning

Hierarchical partitioning was used to determine the independent contribution of the explanatory variables on the occurrence of each of the taxa. The non-numeric variables (i.e. bottom cover, dominant substratum, aquatic and terrestrial vegetation) were excluded from analyses. Data for conductivity, width, depth, elevation and slope were log transformed prior to analyses. Hierarchical partitioning was performed using the ‘hier.part’ package version 1.0-3 [67], which was implemented using the R statistical package version 2.15.1 [62]. Logistic regression and log-likelihood were used as the goodness-of-fit measures in the analyses. Hierarchical partitioning computes the increase in fit for all models containing a given variable, compared to an equivalent model without that variable. The average improvement in fit (i.e. reduction in deviance) across all possible models containing that predictor is then computed. This process results in the estimation of the independent contribution of each explanatory variable (*Ii*), and the joint contribution (*Ji*) resulting from correlation with other variables [50]. The relative independent contribution of each predictor (%*Ii*) can thus be determined. Following Pont et al. [38], a predictor with %*Ii* higher than 100/N (where N in the number of predictors) was considered to have high explanatory power. Therefore, predictors with %*Ii* higher than 14.3% were considered to be important. Randomisation tests which yield *z*-scores were used to determine statistical significance of the relative independent contributions based on an upper confidence limit of 0.95 [50]. Following Chevan & Sutherland [7] and Mac Nally [6], the ratio |*I/J*| was also calculated. Values of this ratio below unit indicate high correlation among predictors.

## Competing interests

The authors declare that there are no competing interests.

## Authors' contributions

This work formed part of AC’s PhD research on the ecology and biogeography of endemic stream fishes from the Cape Floristic Region of South Africa. Both authors contributed to the conception, design and acquisition of data during field surveys. AC analysed the data and drafted the manuscript. Both authors revised and approved the final manuscript.

## Supplementary Material

Additional file 1 Variation in body morphology of the fishes. Click here for file
