# Supplementary material for: Contrasting habitat associations of imperilled endemic stream fishes from a global biodiversity hot spot
Source: BMC Ecol. 2012 Sep 26;12:19. doi: 10.1186/1472-6785-12-19 (PMC3528430; doi:10.1186/1472-6785-12-19)
Supplement: Additional file 1 — Variation in body morphology of the fishes. [file 1472-6785-12-19-S1.docx]

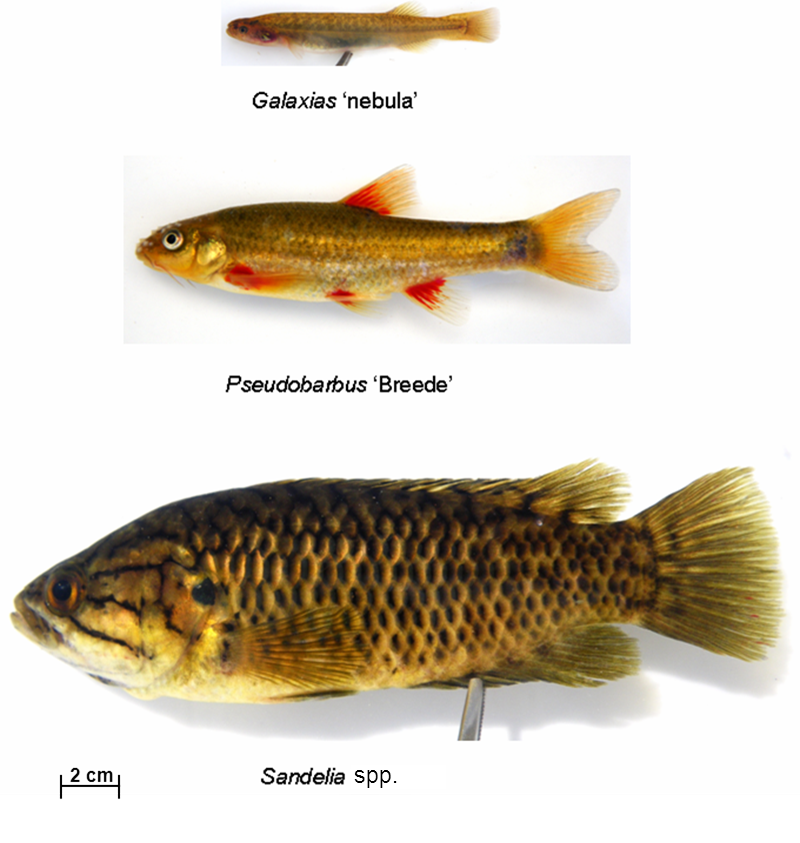


## Appendix 1 – Variation in body morphology of the fishes

Body forms of *Galaxias* ‘nebula’, *Pseudobarbus* ‘Breede’ and *Sandelia* ‘eastern’ scaled to relative adult size.
